# Inhibitory Effects and Composition Analysis of Romanian Propolis: Applications in Organic and Sustainable Agriculture

**DOI:** 10.3390/plants13233355

**Published:** 2024-11-29

**Authors:** Gabriel Heghedűş-Mîndru, Mirel Glevitzky, Ramona Cristina Heghedűş-Mîndru, Gabriela-Alina Dumitrel, Maria Popa, Ioana Glevitzky, Diana Obiștioiu, Ileana Cocan, Mihaela Laura Vică

**Affiliations:** 1 Faculty of Food Engineering, University of Life Science “King Mihai I”, 300645 Timișoara, Romania; gabrielheghedus@usab-tm.ro (G.H.-M.); ramonaheghedus@usab-tm.ro (R.C.H.-M.); dianaobistioiu@usvt.ro (D.O.); ileanacocan@usvt.ro (I.C.); 2 Faculty of Exact Science and Engineering, “1 Decembrie 1918” University of Alba Iulia, 510009 Alba Iulia, Romania; mpopa@uab.ro; 3 Sanitary Veterinary and Food Safety Directorate of Alba County, 510217 Alba Iulia, Romania; ioana_glevitzky@yahoo.com; 4 Faculty of Industrial Chemistry and Environmental Engineering, Politehnica University of Timisoara, 300223 Timișoara, Romania; alina.dumitrel@upt.ro; 5 Department of Cellular and Molecular Biology, “Iuliu Hațieganu” University of Medicine and Pharmacy, 400012 Cluj-Napoca, Romania; mvica@umfcluj.ro

**Keywords:** phyto-inhibitory activity, microbial activity, propolis, cereals, statistics

## Abstract

Propolis is a sustainable and environmentally friendly agrochemical of natural origin, a resinous mixture produced by honeybees. It is used as a natural remedy in multiple pathologies., but it is also a natural defense enhancer, a phytostimulator that helps to bind, bloom, and pollinate plants. Propolis is used in organic farming as a phytoprotector and phytostimulator. The present study investigates the main physical–chemical parameters of Romanian propolis, its antifungal activity against five fungal strains (*Aspergillus niger*, *Aspergillus flavus*, *Penicillium chrysogenum*, *Fusarium oxysporum*, and *Rhizopus stolonifer*) and its phyto-inhibitory activity when it is applied on the layer and under the layer for different grain crops (wheat, maize, oats, and barley). Different doses were used—1, 5, and 10 g of propolis powder—and the growth of the plume was monitored for 13 days. The physical–chemical parameters investigated are volatile oils, wax, oxidation index, melting point, dry matter, ash, and resin, and maximum values were obtained for phenols (189.4 mgGAE/g), flavonoids (84.31 mgQE/g), and IC_50_ (0.086 µg/mL). Propolis demonstrates high antifungal activity against all fungal strains. The results showed that propolis has the best phyto-inhibition potential among the studied grain crops when it is applied on the layer, with the lowest plume growth for maize (14 mm), followed by oats, barley, and lastly wheat (24 mm). Propolis can find increasing application in sustainable and environmentally friendly agriculture and the obtaining of organic food.

## 1. Introduction

Propolis is a natural resinous substance with great potential for use in sustainable agriculture. It is collected by bees from various plant sources, mixed with beeswax and enzymes, and plays an essential role in the construction and maintenance of hives [1,2,3]. Besides these qualities, propolis also contains essential oils with specific aromas that bees collect from different plant species from the outer coverings of pollen grains in the buds of dark green plants, which are brown or light brown, depending on the plant from which it is harvested [4]. For bees, propolis acts as a defense mechanism for hives, supporting their sterility and health. Bees use propolis to immediately embalm what they cannot evacuate from the hive [5]. Therefore, there are significant benefits to human health. The protective properties of the bioactives in propolis make it a potential alternative preventive and therapeutic agent with a broad spectrum of action and make it a kind of natural antibiotic [6,7]. Due to its health benefits, propolis has been used for centuries in traditional medicine [8]. Propolis represents an effective solution for sustainability and environmental friendliness, being used in agriculture as a natural alternative to chemicals, thus contributing to the protection of biodiversity and agricultural ecosystems and maintaining a healthy agricultural environment [2].

The composition of propolis can vary depending on geographic location, plant sources, seasons harvesting, climate changes and bee species [9]. The key components present in propolis are resins (50–70%) that give it its sticky consistency, beeswax (30–50%) secreted by worker bees to create the final texture of propolis, essential oils (10%) and pollen (5%) [10,11]. Essential oils make up some of its aromatic and antibacterial properties. In the rest, there are 5% various organic compounds, namely flavonoids (with antioxidant and anti-inflammatory properties), phenolic acids, aromatic compounds, polysaccharides, enzymes, minerals trace elements, and vitamins [12]. Quercetin, kaempferol, and pinocembrin are abundantly found in propolis [13]. Phenolic acids such as caffeic acid, *p*-coumaric acid, and ferulic acid are compounds with antioxidant and antimicrobial properties present in propolis [14]. Propolis contains aromatic compounds such as benzyl alcohol and benzoyl acetate, which contribute to its characteristic aroma and therapeutic effects [15]. Polysaccharides contribute to the immune system-strengthening properties attributed to propolis [16]. Enzymes in propolis play a role in the decomposition and modification of raw materials [17]. Propolis may contain various minerals and trace elements obtained from plant sources. These include zinc, copper, iron, and manganese [18,19]. Although not in a very large amount, propolis can also contain vitamins such as B complex and vitamins C and E [20].

In many organic certification systems, natural substances such as propolis are accepted, allowing farmers to obtain organic certification for their products. In recent years, the EU strategy on food sustainability protects the environment and confers quality by providing organic products [21]. To avoid the use of pesticides, the natural products obtained by macerating propolis in hydroalcoholic, aqueous solution or used as such in agriculture have become attractive [22,23]. Propolis finds application in agriculture, especially in the management of phytopathogens affecting crops such as tomatoes, coffee, beans, cucumbers, and grapes [24,25,26,27,28,29,30,31]. Propolis can be used as such (in powder form), or its compounds can be selected and extracted in the final solution for the application of treatments [32]. The comparative studies carried out with different organic solvents—methyl alcohol, ethyl alcohol, acetone, propylene glycol, and dimethyl sulfoxide—have revealed different solubilities of different propolis samples taken from different geobotanical regions [33]. Over time, not much research has focused on the use of propolis in agriculture. There are in vitro and greenhouse experiments, and they can bring advantages to agricultural crops. Although the bases of previous studies exist, the practical use of propolis in the field has not been transposed on an industrial scale, requiring the optimization of propolis extracts aimed at its specific use in agriculture [22].

The chemical composition and beneficial properties of propolis also vary depending on the time of collection, but the molecular mechanisms underlying the different biological effects of propolis are still being studied to be elucidated [34]. Propolis from different regions of the world has been extensively studied in terms of its antimicrobial activity, especially against bacteria [35,36,37,38,39]. Some studies have demonstrated the antifungal effect of propolis on species such as *Aspergillus* spp. [40] or *Penicillium* spp., *Fusarium* spp., and *Rhizopus* spp. [41]. Regarding propolis from Romania, some studies have investigated its antimicrobial activity, confirming its antibacterial effect, especially on Gram-positive bacteria, but also on *Candida albicans* [42,43].

The aim of this research is to characterize the physico-chemical properties of propolis from Romania with a view to its integration into agriculture. The objectives of the research are also to test its antifungal activity on strains commonly known to cause grain damage and phyto-inhibitory effects on the growth and development of cereals. The study has potential applications in improving plant-crop management and advancing agricultural practices.

## 2. Materials and Methods

### 2.1. Propolis Samples

The samples were obtained from beekeepers in Romania. Only raw brown propolis was sampled. The raw propolis of *Apis mellifera* bees was harvested from the hive by scraping the wood. Sampling was made by scraping the lid and entering the hives with a stainless-steel spatula. The samples were taken during the same period of the year, between June and July 2023, from 9 historical regions of Romania. A representative sample was taken from each region (from about 20–30 hives belonging to the same beekeeper). The samples were kept at −18 °C in the dark until analysis.

Table 1 shows the number of samples, the geographic region, the county, and the landforms related to the location where the sample was taken.

The choice of the sampling areas (Transylvania, Banat, Crișana, Maramureș, Oltenia, Muntenia, Dobruja, Moldavia, and Bukovina regions) was based on achieving wide coverage of the country and determining the stage of development and the profitability of beekeeping depending on the agricultural crops, the flora, and the relief in which it takes place, thus including all forms.

Meliferous plants produce a lot of nectar and attract bees that use the nectar to make honey. The plants and flora that have the greatest potential to be melliferous in each county under investigation in 2023 are listed in Table 2.

The melliferous plants in Romania, which can produce honey, include sunflower with a yield of 40–100 kg/ha, rapeseed with a yield of 35–100 kg/ha, and several other vegetables and legumes. Alfalfa and clover, both belonging to the legume family, play a crucial role in beekeeping. Bees gather nectar and pollen from the flowers of these plants during their blooming period. The honey yield for alfalfa ranges from 25 to 200 kg/ha, whereas for clover, it ranges from 100 to 250 kg/ha [44].

**Figure 1:**
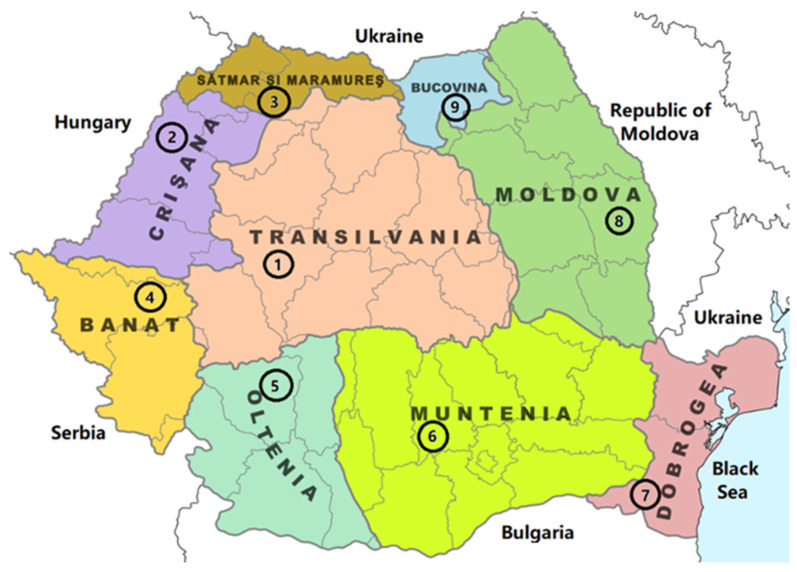
The propolis sampling sites in Romania. 1–9: sampling areas.

Figure 1 displays a map of Romania, indicating the specific counties where the samples were collected within the context of the historical region.

### 2.2. Cereal Samples

Table 3 displays the cereals that have been used in the study to determine the phyto-inhibitory activity of propolis.

The cereals used are from Romania and were harvested in 2023. All the samples were maintained under identical conditions until they were subjected to analysis. The temperature of the grains used was 26.5 °C, a temperature that promotes the key physiological processes of the plants.

### 2.3. Physico-Chemical Analysis

#### 2.3.1. Grain Analysis

The relative mass of 1000 grains: The relative mass of 1000 grains represents the mass of 1000 grains at the moisture they have at the time of determination. Neither the largest nor the tiniest grains are selected while making the determination. G = [(100 − u)/100] g, where: G—absolute mass; u—humidity %; g—mass of 1000 grains in g [45].

The absolute mass of 1000 grains: The absolute mass of 1000 grains represents the weight of 1000 grains relative to the dry substance. To establish the absolute mass, the moisture content is eliminated by calculation, and thus, the absolute mass is calculated according to the formula: G = [(100 − u)/100] g, where: G—absolute mass; u—humidity %; g—mass of 1000 grains in g [45].

The humidity: Grain moisture was determined with Draminski GMM Mini-Grain Moisture Meter (Dramiński, Gietrzwałd, Poland).

Hectoliter seeds mass: The hectoliter mass or volumetric mass represents the mass expressed in kg of a grain volume of 0.1 m^3^ (1 hectoliter).

The glassiness is determined with the Farinotom (Sadkiewicz Instruments, Bydgoszcz, Poland) by cross-sectioning the grain and observing the glassy (transparent) areas and the mealy (matte) areas. Glassiness = 2 × (*n* + 0.75*n*_1_ + 0.5*n*_2_ + 0.25*n*_3_) [%], where: *n* = the number of completely glassy grains, *n*_1_ = the number of three-quarters glassy grains, *n*_2_ = the number of half-glassy grains, *n*_3_ = the number of one-quarter glassy grains [46].

#### 2.3.2. Propolis Analysis

Determination of volatile oils (VO): A distillation flask was filled with 250 mL of distilled water and 50 g of raw propolis that had been crushed. Volatile oils entrained with water vapor were captured in the graduated collector tube, constituting the upper layer. Distillation was carried out until the supernatant volume remained constant [47].

Determination of melting point (MP): A layer of propolis, 3–4 mm in height, was placed by settling into a capillary tube with a diameter of 1 mm, sealed at one end. In the vicinity of the substance layer, a thermometer was fixed. It had paraffin embedded in it. Paraffin was used to embed it. The temperature of the paraffin bath was increased gradually. The temperature at which the material in the capillary tube was completely melted is known as the melting point [48].

Determination of total mineral substances (ash): In a porcelain crucible, 5 g of propolis was added. It was heated in a gas lamp until completely charred. Then, it was calcined for 12 h at 550 °C until at a constant mass. Ash, % = (m_2_ − m)/(m_1_ − m) × 100, where: m—empty crucible mass; m_1_—mass of the crucible with propolis (before calcination); m_2_—mass of the crucible with ash (after calcination) [48,49].

Determination of wax content: The process of determining the extractable substances (wax) in n-hexane involved weighing 2 g of sample placed in a cellulose cartridge. Then, using the Soxhlet technique, condensation was performed from a 200 mL volume of n-hexane for 6 h. The volume remaining in the ball was then measured, and 10 mL was taken and dried at 105 °C. The weight of the dried wax was used to estimate its proportion in the total sample [47].

Determination of the oxidation index (OI): A total of 0.1 g of raw propolis is added to 2.5 mL of ethanol. After one hour, 50 mL of water was added, stirred (250 rpm), and filtered on Whatman No. 3 filter paper (Whatman, UK). To 2 mL of this filtrate, add 20% sulfuric acid solution and a drop of 0.1 N potassium permanganate. Record with a stopwatch the time, in seconds, that the solution takes until the discoloration has been recorded. The timer was started while constantly shaking. The insoluble residue retained in the cellulose thimble at the end of the Soxhlet extraction is dried in an oven at 80 °C to constant weight [50].

Determination of resin. The sample resulting from the extraction of extractables with hexane was placed in the body of a Soxhlet extractor for further extraction with ethanol. Finally, the ethanol content was brought to a volume of 100 mL. An aliquot was taken and placed on plates and dried at 80 °C, and the dry wax weight was used to estimate its proportion in the total sample [47].

Total phenolic content (TPC)—Folin–Ciocalteu method: A total of 50 g of dried raw propolis was dissolved and homogenized in 15 mL ethanol. The mixture was filtered with Whatman No. 1 filter paper. An equivalent quantity of Folin–Ciocalteu reagent was added. The absorbance was measured against a blank (distilled water) at 765 nm with a Lambda 20 UV–VIS Spectrophotometer (Perkin Elmer UV/VIS, Washington, DC, USA). The total phenolic concentrations were compared to a standard curve of gallic acid [51,52].

Total flavonoid content (TFC): A total of 2.5 mL of 96% ethanol was added to 1 g of propolis, and the mixture was centrifuged for 24 h at 200 rpm with a Centra CL2 centrifuge (Thermo Fisher Scientific Inc., Waltham, MA, USA), after which 25 mL of 80% ethanol were added. A total of 0.1 mL Folin–Ciocalteu reagent 10%, 0.1 mL CH_3_COO-K 1M, 1.5 mL ethanol 95%, and 2.5 mL distilled water was then added. The mixture was kept in the dark for 50 min. Absorbance was measured at 425 nm Lambda 20-Perkin Elmer UV/VIS Spectrophotometer (Waltham, MA, USA). TFC (mg quercetin equivalents (QE)/100 g propolis) was calculated using a calibration curve with a standard quercetin solution [53].

Antioxidant activity (AA): DPPH (2,2-diphenyl-1-picrylhydrazyl) (Sigma-Aldrich, St. Louis, MO, USA) was used as free radical for the evaluation of propolis radical scavenging activity (RSA). An alcoholic extract of propolis was prepared at room temperature by homogenizing propolis with 70% ethanol solution (1:100 *w*/*v*) for 24 h. After the complete evaporation of the alcohol, the concentrated dry substance is used. Two solutions were prepared: 0.6 mg/mL propolis and DPPH 0.1 mM ethanolic solution.

The absorbance was measured at λ = 515 nm with a Lambda 20 UV–VIS Spectrophotometer (Perkin Elmer UV/VIS, Washington, DC, USA). The absorbance (A) was measured at the initiation of the reaction, then after 10 and 20 min. The antioxidant activity was calculated using the formula: %RSA = (A_DPPH_ − A_sample_)/A_DPPH_ × 100 [54,55]. The free-radical scavenging capacity of propolis samples was expressed as the IC_50_ value. To establish the IC_50_, the RSA value was determined for each propolis sample at 5 different concentrations: 0.6, 1, 2, 3, and 4 mg/mL.

#### 2.3.3. Obtaining the Aqueous Extract of Propolis

The aqueous propolis extracts were obtained as described in our previous study [56,57]. Briefly, aqueous suspensions of propolis powder were obtained (50 g of purified, finely divided propolis with 250 mL of distilled water) and refluxed for one hour, after which they were centrifuged twice at 12,000 rpm and 10,000 rpm, respectively. To evaporate 80% of the initial mixture, they were held at the boiling point of water. The aqueous extracts of propolis thus obtained were stored in a dark and dry space until use.

#### 2.3.4. HPLC Analysis

High-performance liquid chromatography analyses were performed using Hewlett Packard Agilent 1100 HPLC System with UV detection (Marshall Scientific, Hampton, NH, USA). Chromatographic separation was accomplished using a stainless-steel analytical column—Nucleosil C18 column from Macherey–Nagel (Düren, Germany) with a stationary phase particle size of 5 μm (4.6 × 150 mm). The column flow rate was set at 1 mL/min. Sample injections of 20 µL were used for all samples and standards. The eluent consisted of acetonitrile and water in a 1:1 ratio, with detection at a wavelength of 365 nm and a temperature of 30 °C. Aqueous propolis extracts were initially dissolved in ethanol (5 mg/mL), filtered through a 0.45 μm filter, and then 20 µL was injected into the HPLC system.

Reflux extraction was performed for all propolis samples according to the method described in Section 2.3.3. The main content of flavonoids, quercetin and rutin (compounds with the most studied biological activity), was quantified using the HPLC method. For each extract, five consecutive replicates were conducted, with relative standard deviations (RSD) subsequently calculated.

### 2.4. The Phyto-Inhibitory Activity of Propolis

The phyto-inhibitory activity is based on the estimation of the period of slowing down the germination of cereal samples with physico-chemical characteristics in standard systems, with and without the addition of propolis.

In Petri dishes (with a surface area of 20 cm^2^) on which the same amount of hydrophilic wool was introduced, cereal grains were added and watered periodically. Later, 1, 5, and 10 g of propolis (powder) were introduced. The two cases followed were: (1) homogenization of propolis with the wool layer (in layer); and (2) propolis was added to the surface of hydrophilic wool (on layer). Readings were taken every other day for 13 days, and statistical evaluations (averages) were performed on 10 sprouted seedlings [56,57].

### 2.5. Antifungal Activity of Propolis

The aqueous propolis extracts, prepared at a concentration of 0.1 g/mL, were used to evaluate the antifungal activity.

#### 2.5.1. Fungal Cultures

The antifungal efficacy of aqueous propolis extracts was assessed using five fungal strains commonly known to cause grain damage: *Aspergillus niger* (derived from ATCC 16888), *Aspergillus flavus* (ATCC 9643), *Penicillium chrysogenum* (ATCC 10106), *Fusarium oxysporum* (ATCC 48112) and *Rhizopus stolonifer* (ATCC 14037), provided by MicroBioLogics Inc. (St. Cloud, MN, USA) and Thermo Fisher Scientific Inc. (Waltham, MA, USA). To obtain cultures, 3–5 colonies from each fungal strain were dispersed in 10 mL Sabouraud Dextrose Broth (SDB) (Merck KgaA, Darmstadt, Germany) and were incubated for 72 h at 25 °C. To obtain cell suspensions for testing, the turbidity was adjusted to 0.5 McFarland using a McFarland densitometer (Mettler Toledo, Columbus, OH, USA).

#### 2.5.2. Antifungal Properties of the Aqueous Propolis Extracts—Agar Disk-Diffusion Method

According to CLSI-recommended procedures [58], the disk-diffusion method was used by measuring the diameters of the inhibition zones produced by fungal strains. The diameter of the inhibition zone was considered to be a semi-quantitative measure of the antifungal activity. The culture medium Sabouraud 4% dextrose agar (Merck KGaA, Darmstadt, Germany) in Petri dishes, with a depth of ~4 mm, was inoculated by flooding with 1 mL fungal culture. Filter paper discs (with a diameter of 6 mm) impregnated with 50 µL of each propolis aqueous extract (0.1 g/mL concentration) were placed on agar, and the Petri dishes were incubated for 5 days at 25 °C. Discs containing 1 μg voriconazole (Bio-Rad, Marnes-la-Coquette, France) were used as a positive control. All tests were performed in triplicate by the same operator and under the same laboratory conditions. The inhibition zone diameters (in mm) were measured using a DIN 862 ABS digital caliper (Fuzhou Conic Industrial Co., Ltd., Fuzhou, China).

#### 2.5.3. Minimal Inhibitory Concentration (MIC)

The values of the minimal inhibitory concentration (MIC) were carried out using the micro-dilution broth method, according to the European Committee on Antimicrobial Susceptibility Testing (EUCAST) [59].

Therefore, 5 μL of each propolis extract was added to the first line of the microtiter wells containing 195 μL SDB, followed by 2-fold serial dilutions with final concentrations ranging from 25 µg/mL to 0.19 µg/mL for all propolis extracts. A total of 100 μL volumes of the suspensions with fresh microbial cultures were inoculated into wells containing the propolis extracts. The MIC results were recorded as the lowest concentration of propolis extract that inhibits the visible growth of a microorganism. A negative control (containing the tested extracts without the inoculum) was included on each microplate.

#### 2.5.4. Minimal Fungicidal Concentration (MFC)

The minimal fungicidal concentration (MFC) values were determined by inoculating 10 µL of inoculum from the wells where the inhibitory effect was observed onto the Sabouraud 4% dextrose agar plate. The plates thus prepared were incubated for 5 days at 25 °C, and fungal growth was observed. The MFC was determined for the lowest dilution at which fungal growth was blocked.

### 2.6. Statistical Analysis

For the germination periods of the cereal samples treated with different doses of propolis on the layer and under the grain crops layer, principal component analysis (PCR) was used with the software Origin Pro 2020 (Stat-Ease Inc., Minneapolis, MN, USA), respectively the Pearson correlation coefficient to correlate the content of phenols and flavonoids with the MIC of microorganisms. The Pearson’s correlation test was performed using Minitab software (Minitab, LLC, State College, PA, USA).

## 3. Results

### 3.1. Physico-Chemical Analysis of the Propolis Samples

The average values of the physical–chemical parameters of the propolis samples collected from all regions of Romania are presented in Table 4 and Table 5.

The physical–chemical characteristics of Romanian propolis exhibit regional variations in the results obtained. Volatile oils range in concentration from 0.1 to 0.5%. The range of values for the wax content is 24.54 to 30.02%. Samples of propolis had an oxidation index ranging from 10.7 to 14.8 s. For propolis samples, the melting point is between 62 to 66 °C, which is rather close. The ash concentration varies between 0.79 to 2.01%, whereas the dry matter values range from 3.27 to 3.74%.

Notable differences are found between propolis samples from the whole country. Even if these variations are small, one of the most important characteristics of propolis is its antioxidant activity, and phenolic compounds and flavonoids act as an indirect measure of antioxidant activity. In the case of the TPC, the values are between 102.7 (S6—Dâmbovița) and 189.4 mg (S1—Alba) of GAE/g. The TFC varies between 65.30 (S6—Dâmbovița) and 85.19 mg (S7—Constanța) of QE/g. The IC_50_ value in the DPPH assays showed a high antioxidant capacity of Romanian propolis. A lower IC_50_ value, such as Sample 7 from Constanța County, means a higher antioxidant activity. To correlate the physico-chemical parameters with each other for the 9 propolis samples, the intercorrelation matrix for the correlation coefficients was generated. The results are presented in Table 6.

It is observed that the best linear correlation is found between the oxidation index and the TPC of propolis samples. Our results showed a significant negative correlation between the total phenolic content in propolis samples and their IC_50_ for DPPH scavenging activities. This indicates that higher total flavonoid or phenolic content corresponds to a lower IC_50_ for DPPH scavenging activity value, which, in turn, signifies stronger antioxidant activity. No significant correlation between total flavonoid content and IC_50_ values for DPPH can be attributed to the fact that not all flavonoids exhibit strong antioxidant properties. The Pearson correlation coefficient indicates a strong intensity of the relationship between the dry matter and wax content parameters (r = 0.825). Many of the parameters studied exhibit a moderately high correlation, such as TFC and the content of volatile oils, oxidation index, and TPC (with r between 0.616 and 0.637). Appreciable relationships were also found between TPC and resin content (r = 0.683). A moderate positive correlation exists between the ash content and the content of volatile oils and between resins and oxidation index. These satisfactory intercorrelations were recorded as r = 0.519 and r = 0.595. Otherwise, a negative correlation was found between most of the parameters.

### 3.2. HPLC Analysis

Most studies have used alcoholic extracts of propolis, with few focusing on the biological activities of aqueous propolis extracts. In a previous study [60], we compared the efficiency of ethanolic extracts (at different concentrations) with the aqueous one in the quantification of some flavonoids in order to better understand the balance between efficacy and toxicity, which is essential for a natural food supplement or phytosanitary product, in the present study the characteristics were investigated only of the aqueous extract of propolis.

**Figure 2:**
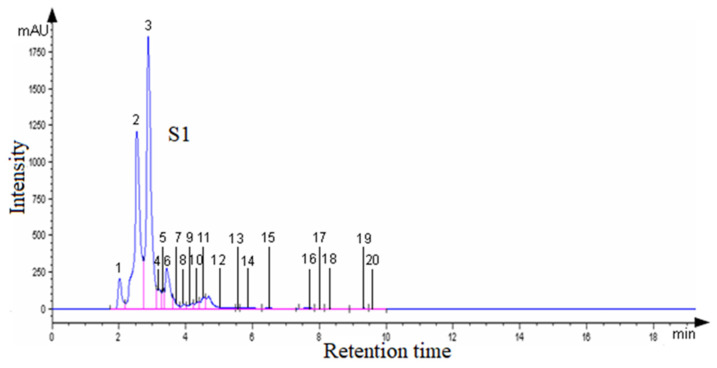
The HPLC chromatogram of S1 (Alba County) aqueous propolis extract.

Figure 2 shows the HPLC detection of the active compounds in the aqueous extract of propolis S1, sampled from Alba County.

In the aqueous propolis extract from Alba County (S1), 20 compounds were separated and quantified using the area method: quercetin (ketonic form) 1.47%, quercetin (enolic form) 7.37%, and rutin 3.94%. The retention times and peak areas of the active substances in propolis are presented in Supplementary Table S1. In the aqueous extract of propolis S1, along with rutin and quercetin, the following compounds were identified: caffeic acid (34.16%), p-coumaric acid (40.71%), 3,4-dimethoxycinnamic acid (2.44%), apigenin (0.87%), kaempferol (1.92%), galangin (0.21%), phenethyl caffeate (0.20%), and cinnamate (0.20%).

The major components in the aqueous propolis extract samples were identified through RP-HPLC analysis. For quercetin and rutin, standards were used, while the other components were identified by comparing the obtained HPLC chromatograms under the same conditions to those found in the literature.

**Figure 3:**
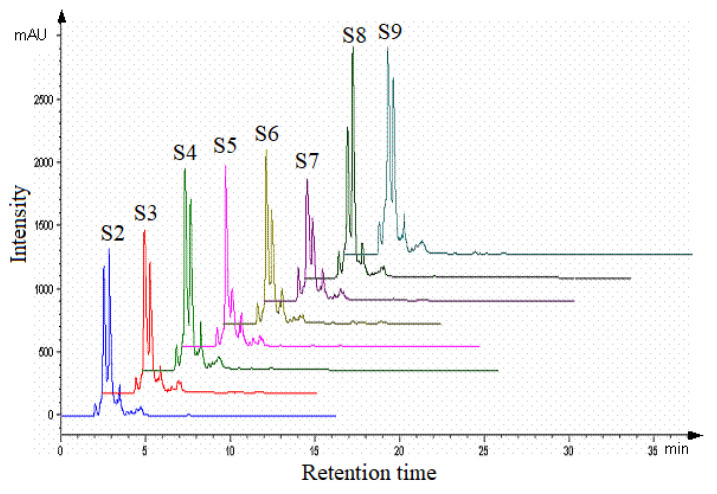
Comparative HPLC chromatograms of Romanian propolis aqueous extracts S2–S9.

In Figure 3, a comparative evaluation, delayed by 10 min, of the chromatograms for the aqueous propolis extracts S2–S3 from Romania is presented.

The aqueous propolis extract S2 from Bihor County allowed the identification of 18 compounds (by comparing the retention times of rutin and quercetin standards and by comparing the HPLC chromatograms obtained under the same conditions with those in the literature). These are rutin (2.12%), caffeic acid (35%), p-coumaric acid (41.79%), 3,4-dimethoxycinnamic acid (1.15%), quercetin (ketonic form) (2.02%), quercetin (enolic form) (9.81%), apigenin (1.24%), kaempferol (1.82%), galangin (0.06%), phenethyl caffeate (0.08%), and cinnamate (0.20%).

In the aqueous propolis extract S3 (Maramureș County), 18 compounds were identified, namely: rutin (2.99%), caffeic acid (41.74%), p-coumaric acid (33.15%), 3,4-dimethoxycinnamic acid (1.31%), quercetin (ketonic form) (1.67%), quercetin (enolic form) (8.24%), apigenin (1.38%), kaempferol (0.40%), galangin (0.19%), phenethyl caffeate (0.36%), and cinnamate (0.66%).

For the aqueous propolis extract S4 (Timiș County), 16 compounds were identified with the following proportions: rutin (3.45%), caffeic acid (40.85%), p-coumaric acid (30.91%), 3,4-dimethoxycinnamic acid (2.04%), quercetin (ketonic form) (1.85%), quercetin (enolic form) (9.9%), apigenin (1.94%), kaempferol (6.52%), galangin (0.25%), phenethyl caffeate (0.34%), and cinnamate (0.53%).

In the case of the aqueous propolis extract S5 from Gorj County, 18 compounds were identified: rutin (3.41%), caffeic acid (51.80%), p-coumaric acid (18.79%), 3,4-dimethoxycinnamic acid (1.20%), quercetin (ketonic form) (3.95%), quercetin (enolic form) (9.3%), apigenin (1.70%), kaempferol (2.26%), galangin (0.28%), phenethyl caffeate (0.39%), and cinnamate (0.89%).

For the aqueous propolis extract S6 (Dâmbovița County), 20 compounds were identified, with rutin extracted at a proportion of 3.57%, caffeic acid (52.11%), p-coumaric acid (17.08%), 3,4-dimethoxycinnamic acid (1.58%), quercetin (ketonic form) (3.02%), quercetin (enolic form) (8.7%), apigenin (1.51%), kaempferol (2.90%), galangin (0.25%), phenethyl caffeate (0.36%), and cinnamate (0.83%).

The HPLC analysis of the aqueous propolis extract S7 (Constanța County) allowed the identification of 18 compounds, namely: rutin (3.42%), caffeic acid (41.18%), p-coumaric acid (32.11%), 3,4-dimethoxycinnamic acid (1.12%), quercetin (ketonic form) (3.13%), quercetin (enolic form) (9.07%), apigenin (1.35%), kaempferol (1.87%), galangin (0.21%), phenethyl caffeate (0.43%), and cinnamate (1.07%).

In the aqueous propolis extract S8 (Vaslui County), 19 compounds were identified: rutin (6.93%), caffeic acid (40.9%), p-coumaric acid (26.48%), 3,4-dimethoxycinnamic acid (1.34%), quercetin (ketonic form) (1.97%), quercetin (enolic form) (9.91%), apigenin (1.40%), kaempferol (3.63%), galangin (0.16%), phenethyl caffeate (0.45%), and cinnamic caffeate (0.56%).

In the aqueous propolis extract S9 (Suceava County), 17 compounds were identified: rutin (4.31%), caffeic acid (44.28%), p-coumaric acid (29.15%), 3,4-dimethoxycinnamic acid (1.53%), quercetin (ketonic form) (1.66%), quercetin (enolic form) (8.19%), apigenin (1.26%), kaempferol (6.55%), galangin (0.20%), phenethyl caffeate (0.31%), and cinnamate (0.43%).

Table 7 presents the concentrations of quercetin and rutin in the nine aqueous extracts of propolis.

The analysis of propolis samples collected from Romania, conducted using HPLC, identified the presence of two flavonoids: quercetin and rutin. When quantifying quercetin in aqueous extracts (based on the calibration curve), concentrations were recorded, ranging from 0.62 to 0.81 mg/mL, and routine values were between 0.0093 to 0.0168 mg/mL depending on the geographical area where it was taken.

The behavior of aqueous propolis extracts from the point of view of antioxidant, microbiological, and phytoncide activity can be explained based on the relative concentration of the compounds with such action from the analyzed extracts (quercetin and rutin). Under the conditions of determination, they separate at retention times of 3.1 and 3.4 min and at 2.1 min. The HPLC analysis of quercetin indicated the appearance of two more important components, most likely due to the 3-enolic and 3-ketonic boundary structures that are in equilibrium in the ethanolic quercetin solution.

### 3.3. Physico-Chemical Analysis of the Cereals

To assess the quality of cereals, the following parameters were determined: relative weight of 1000 seeds, absolute mass of 1000 seeds, moisture, hectoliter mass, and glassiness. The results of the analyses obtained for the physical characteristics of the grains used are presented in Table 8.

The mass of 1000 seeds is influenced by the specific masses, the proportions of the anatomical parts of the grain, and their humidity. The humidity of propolis is influenced by both the handling conditions and the storage time.

### 3.4. The Phyto-Inhibitory Activity of Propolis

**Figure 4:**
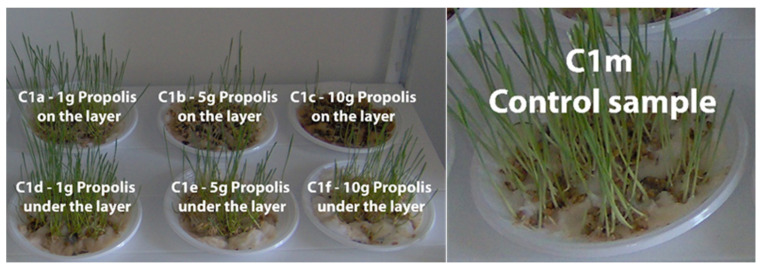
Wheat samples (C1) after 13 days in the comparative evaluation of the phyto-inhibitory activity of propolis on the layer and under the layer.

**Figure 5:**
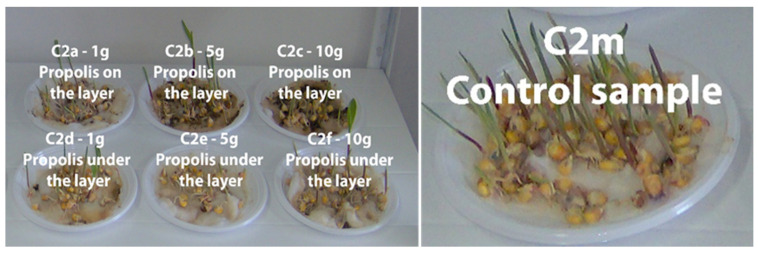
Maize samples (C2) after 13 days in the comparative evaluation of the phyto-inhibitory activity of propolis on the layer and under the layer.

**Figure 6:**
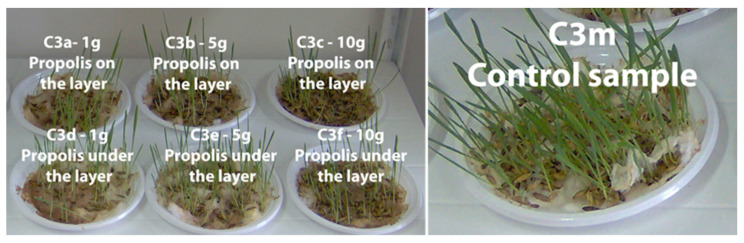
Oat samples (C3) after 13 days in the comparative evaluation of the phyto-inhibitory activity of propolis on the layer and under the layer.

**Figure 7:**
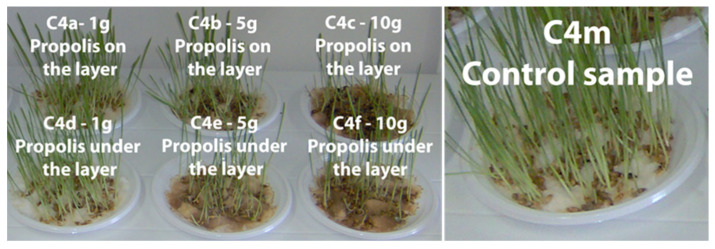
Barley samples (C4) after 13 days in the comparative evaluation of the phyto-inhibitory activity of propolis on the layer and under the layer.

In Figure 4, Figure 5, Figure 6 and Figure 7 the plume of the cereal samples is presented comparatively after 13 days by applying propolis on the layer and under the layer, and the control sample.

Supplementary Tables S2, S4, S6 and S8 present plumule growth lengths (mm) for the C1, C2, C3, and C4 samples treated with different amounts of propolis powder under the layer. Comparatively, in Supplementary Tables S3, S5, S7 and S9, plumule growth lengths (mm) are presented for the same samples treated with different amounts of propolis powder but per layer in time and the control sample (M).

It is shown that in terms of the average plume development lengths for the wheat samples, the amount of propolis applied clearly correlates with the gradual slowing down of plant growth. After 13 days, Sample S4 from Timiș County and Sample S9 from Suceava have the lowest growth at 1 g and 10 g, respectively. Additionally, sample S4 exhibits the lowest level of plume growth at the end of the monitoring period after applying 5 g of propolis. Except for the samples where 10 g of propolis was applied, the radicle and plume of the wheat samples are evident from the very first few days. The control sample grows at the fastest rate since it does not include propolis as an inhibitor.

The tendency of plume growth in maize samples treated with propolis powder is similar to that of wheat samples. Samples S7 (Constanța County) and S9 (Suceava County), which received 1 g of propolis, and samples S8 (Vaslui County) and S9 (Suceava County), which received 10 g of propolis, have the smallest increases of the plume.

When 1 g or 5 g of propolis powder is applied as an inhibitor to the maize samples, the plume growth values are similar; nevertheless, for all 9 propolis samples, the difference from the control sample is not very significant. The plume growth rate is much slower when 10 g of propolis powder is applied to the maize sample.

When 10 g of inhibitory propolis is applied to oat samples, Sample S3 (Maramureș County) and Sample S4 (Timiș County) show the smallest average increase in plume lengths after 13 days.

The growth rate of the barley seed plume at doses of 1 g and 5 g did not significantly differ among the propolis samples that were gathered from various historical regions of Romania. When 10 g is applied, these become more noticeable. Sample S4 (Timiș County) has the lowest growth, while Sample S2 (Bihor County) has the most development. The biggest difference between the two samples is 11 mm.

### 3.5. Antifungal Activity of Propolis

Table 9 shows the inhibition zone diameters (measured in mm) of nine propolis samples from various regions of Romania that were assessed against specific fungal strains.

As shown in Table 9, all propolis extracts showed antifungal activity against all tested strains, with the diameters of the inhibition zones varying between 15 and 28 mm. In the case of the *R. stolonifer* strain, all propolis samples had larger diameters of the inhibition zones than the antifungal agent, and for the *P. chrysogenum* strain, 4 samples (S1, S3, S5, and S7) had a stronger effect than this antifungal.

The most sensitive strains to the effect of propolis were those of *F. oxysporum* and *R. stolonifer*, followed by *A. flavus*. Samples S1 and S3 were the most effective regarding the antifungal effect, presenting the largest diameters of the inhibition zones.

Regarding MIC, except for Sample S4, all samples had an antifungal effect up to concentrations of 6.25 mg/mL, but not for all strains. Many of the samples had an inhibitory effect at concentrations of 3.12 mg/mL on some of the strains, and Sample S1 had an effect on the strains of *F. oxysporum* and *P. chrysogenum* even at concentrations of 1.56 mg/mL.

The fungicidal effect (MFC) was observed for all samples at the concentration of 12.5 mg/mL, and, for Sample S1, MFC was observed at the concentration of 6.25 mg/mL on the strains of *F. oxysporum* and *P. chrysogenum*.

A Pearson correlation of each propolis sample’s total phenol and flavonoid content with their MIC was performed and presented in Table 10.

As can be seen from the statistical relation between MIC and polyphenols concentration in Table 10, the correlations between MIC and phenols and flavonoids were low for the microorganisms studied and statistically insignificant (except for *P. chrysogenum*, where r = 0.731). The low correlation may result from the complex nature of propolis, including its varied composition, specific interactions between its components and microorganisms, possible synergistic or antagonistic effects, and the presence of non-phenolic or non-flavonoid compounds with strong antimicrobial properties. However, correlation coefficients of 0.600 (in the case of TPC and *F. oxysporum*) and 0.667 (in the case of TFC and *A. niger* or *F. oxysporum*) indicate appreciable correlations.

## 4. Discussion

Considering the greater interest in the use of natural components in different fields of agriculture and industry, investigating the composition of propolis as a sustainable rural and agricultural tool is necessary. The physico-chemical results of the Romanian propolis samples indicate that they have different quantities of resin, wax, balsam, oxidation index, or melting point, with significant differences between them depending on the geographical area. The analyzed propolis is in accordance with the values obtained by Okińczyc et al. [61] for Polish propolis in the range of 0.07% to 2.8% for essential oils and wax 20-30% (which affects its texture and solubility). The average value for the wax content is 27.75% in agreement with what is reported in one study conducted on Romanian propolis [50]. The highest value is recorded in Sample S7 (Constanța) and S4 (Timiș), and the lowest in S3 (Maramureș). Essential oils do not contain any substance that can be used as a strong marker of propolis origin. However, the role of essential oils as a secondary marker cannot be excluded because of the attraction role of some of the monoterpenes, sesquiterpenes, and aromatic esters [62]. The ash content highlights the presence of inorganic minerals, as well as the presence of impurities present in the sample, such as wood, bee droppings, and soil. The analyzed samples have an average ash content of 1.22%. These results are those obtained by El-Guendouz et al. and Grassi et al. [63,64], where the values are between 0.72 and 5.01%. The scientific literature analysis [15] reveals the same amount of ash without major differences between the analyzed samples from Romania.

Regarding European propolis from temperate zones, its biological activities are generally given by the main constituents: flavonoids and phenols (quercetin, kaempferol, pinocembrin, caffeic acid, *p*-coumaric, rutin, ferulic acids etc.). The results showed that the total phenolic and flavonoid contents were in the range of 102.7–189.4 mg GAE/g and 65.3–85.19 mg QE/g, which contribute to the major component of propolis bioactive properties of propolis. Similar research shows that Turkish propolis contains between 16.73 and 98.89 TPC and 57.98 and 327.38 mg QE/g TFC [14], and propolis from Mexico has values of flavonoids from 13 to 379 mg and phenols from 68 to 500 mg per g of propolis [65].

In general, the lower the IC_50_ values, the more effective the substance is at scavenging DPPH and indicates a strong antioxidant activity if the IC_50_ value obtained is <50 ppm. The observed IC_50_ value showed that Sample S7 from Constanța County exhibited the highest antioxidant activity (0.086 µg/mL), followed by Sample S1 from Alba County (0.333 µg/mL). The total phenolic and flavonoid content of the propolis samples had a significant negative correlation with their IC_50_ scavenging activities for DPPH. Phenols and flavonoids are known as good scavengers due to their ability to donate electrons [66].

Regarding the phyto-inhibitory effect of propolis, it represents its ability to inhibit the germination of seeds and the growth of plant roots. Flavonoids and phenolic acids are the main bioactive compounds [48], which can interfere with plant physiological processes. Relevant literature provides information about propolis used as a herbicide. Sorkun et al. [67] investigated the phyto-inhibitory or phytotoxic effect of propolis. Another study by Sorkun et al. [68] provides information on seed germination delay in plants treated with propolis extracts. Propolis can prevent or delay the germination of weed seeds, namely broad leaf seed germination, better than narrow leaves. Our results coincide with the statement of Dadgostar and Nozari [69], as by increasing the concentration of the propolis solution, its effect changes.

In our study, regardless of the dose applied—1, 5, or 10 g—the propolis samples could inhibit or slow down the growth of the plumes of the analyzed cereals. Propolis placed under the layer leads to increases in the plume growth lengths in all crops. The highest value is observed when a dose of 1 g of propolis is applied, and it lowers as more propolis is added.

King-Díaz et al. [70] demonstrated that due to the content of flavonoids, propolis showed activity on the growth of seedlings of *Lolium perenne* and *Echinochloa crus-galli*. Fernandes-Silva et al. [71], through their study, showed that the essential oil of propolis influences both the germination of seed lettuce and the growth of its seedlings, manifesting a phytotoxic potential.

The role of propolis is to protect the hive, cover the corpses of pests (insects and arthropods), and defend against microbial pathogens [48]. Therefore, propolis also has an insecticidal role with direct effects on the reproduction of the ectoparasite, Varroa destructor, and the small wax moth, *Achroia grisella* [72,73]. Propolis is a natural plant strengthener, accepted in organic agriculture, which protects against fungal attacks [74].

Propolis is applicability in the case through the process of conversion and ecological certification for a vegetable farm [75]. It can be used alone or in combination with common fungicides to synergistically exploit the raw material. Due to its low solubility and high content of resinous matter and essential oils, it is more difficult to be washed away by rain and has a prolonged functionality within crops [76]. Yang et al. [77] showed the effect of propolis as a natural antifungal agent to control citrus blue and green mold.

In the case of wheat treated with propolis on the layer, this powder forms a fine, flexible film, which has a protective and phyto-inhibitory effect on plant growth. It is recognized that propolis is used in the cosmetic industry due to its content of active components with antimicrobial, cicatrizing, and epithelializing properties [78]. Similarly, in the growth of grains, propolis supports the barrier function and limits bacterial development, preventing the penetration of pathogens at the same time. Thus, the average lengths of plume growth are much lower than in the case of applying propolis in a layer.

The values obtained in our study align and follow the same general pattern as the previous research [79,80]. When compared to 1 and 10 g doses, the plume growth values at 5 g are intermediate. The growth of the plume is proportionally inhibited by increasing the propolis dose. In the current study, we also chose to determine the antifungal effect of propolis samples on some species selected from the main fungal species found on cereals and compare its effect with that of an antifungal agent (voriconazole). The antimicrobial activity of propolis has been studied so far using alcoholic or aqueous extracts of this bee product [81]. Unlike other studies that investigated alcoholic extracts of propolis from Romania [42], our study used aqueous extracts of propolis (to avoid the influence of ethanol on its antifungal properties) and managed to demonstrate its antimicrobial activity. A strong antifungal activity against *F. oxysporum* and *R. stolonifer* species was demonstrated, in the case of *R. stolonifer* stronger than the antifungal used as a control. In the case of these strains, MIC and MFC were also the lowest, confirming the strong antifungal effect of propolis. The results obtained also confirm our previous results regarding the antifungal effect of propolis from Transylvania [82] or Romania [80].

Table 10 shows the correlations between the MIC and the concentration of polyphenols, and that of flavonoids for all microorganisms studied. The correlations between MIC and flavonoids and MIC and phenols were relatively low and statistically insignificant for the microorganisms studied. However, the exception is the strength of association in the case of MIC for *A. niger, P. chrysogenum,* and *F. oxysporum* with the content of TPC and TFC of a propolis sample, which presents an even stronger positive correlation. Flavonoids have long been known for their frequent use in the treatment of various human diseases. Flavonoids are already known to inhibit fungal growth through underlying mechanisms such as plasma membrane disruption, induction of mitochondrial dysfunction, and inhibition of cell wall formation, cell division, RNA and protein synthesis, and the efflux-mediated pumping system [83]. Also, phenols act on fungi through mechanisms such as the downregulation of some metabolic pathways, the induction of apoptosis, inhibitors of some biosynthesis pathways, the inhibition of the activity of certain enzymes, or the reduction of the adhesion properties of the fungus [84]. In the current study, through the positive linear relationship between MIC and these compounds, it was confirmed that these propolis components determine the antifungal effect.

Considering the growing interest in using natural components across various fields of agriculture and industry, propolis as a sustainable agricultural tool is essential, as these data support its phyto-pharmaceutical potential and highlight its application in plant-crop management as an alternative in organic agriculture.

**Figure 8:**
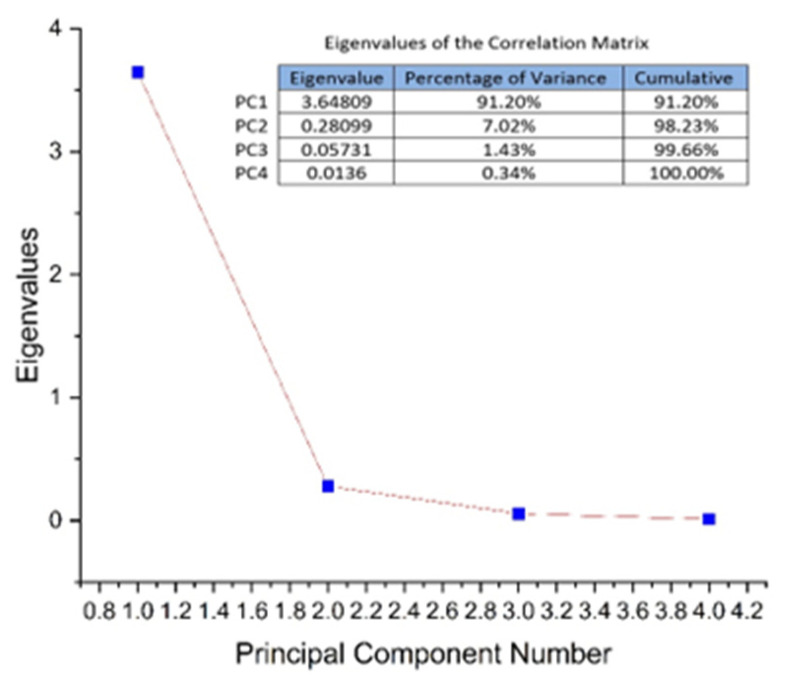
The total variance of the PCs and the eigenvariance for each of the PCs if the propolis is applied under the layer.

PCA multivariate data analysis is a mathematical method that achieves data dimensionality reduction and allows visualization of the underlying structure in experimental data as well as relationships between data and samples. The analysis (PCA) aimed to evaluate the phyto-inhibitory effect of propolis applied under the layer, in amounts of 1 g, 5 g, and 10 g, on the four cereal samples after 3, 5, 7, 9, 11, and 13 days. As input data (variables), the growth lengths of the grain samples were used depending on the period and the amount of solid propolis applied under the layer. Determination of principal components was performed based on the values of the correlation matrix. The obtained eigenvalues were from 3.64, 0.28, 0.057, and 0.013 for PC1 to PC4. As can be seen in Figure 8, the first three PCs explain 99.66% of the total variance of the data. PC1 explains 91.20%, PC2 explains 7.02% and PC3 explains 1.43%.

**Figure 9:**
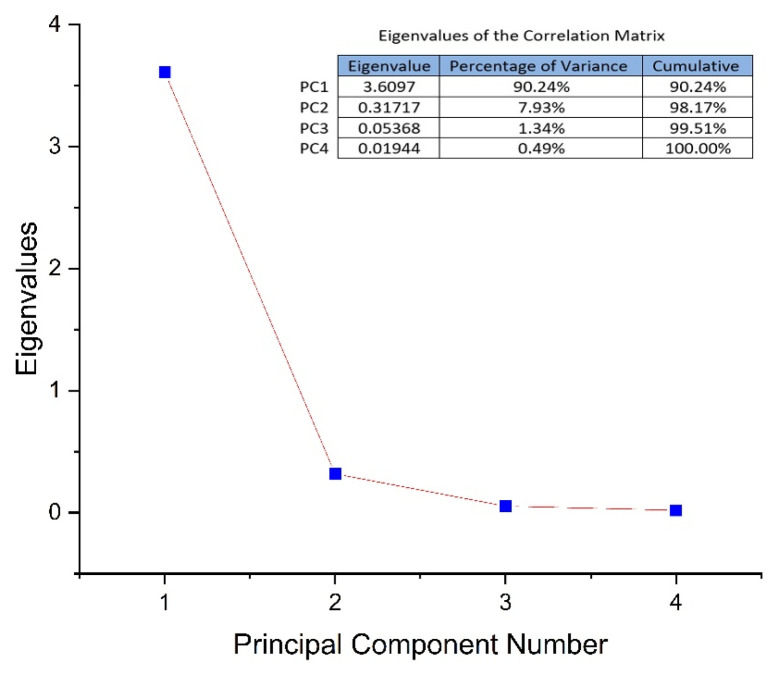
The total variance of the PCs and the eigenvariance for each of the PCs if the propolis is applied on the layer.

Comparatively, the multivariate PCA evaluation was performed using, as input data (variables), the growth lengths of the grain samples depending on the period and the amount of solid propolis applied on the layer. The obtained eigenvalues were from 3.60, 0.31, 0.05, and 0.01 for PC1 to PC4. As can be seen in Figure 9, the first three PCs explain 99.51% of the total variance of the data. PC1 explains 90.2%4, PC2 explains 7.93% and PC3 explains 1.34%.

**Figure 10:**
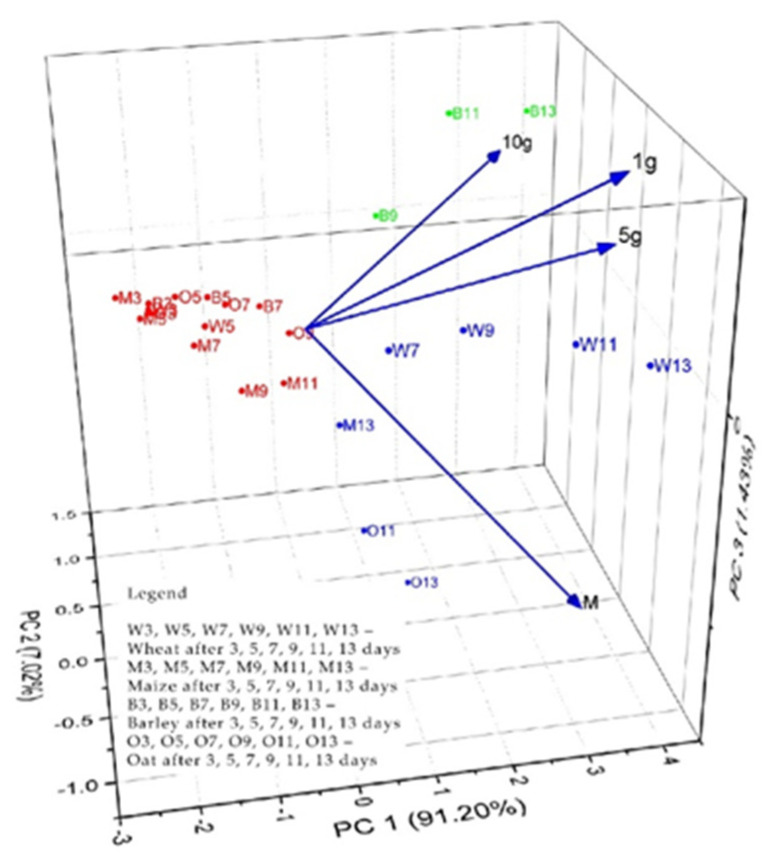
Biplot scores and loadings 3D for PCA analysis of plume evolution of cereal samples treated under the layer with 1 g, 5 g, and 10 g solid propolis over a period of 3, 5, 7, 9, 11, and 13 days.

**Figure 11:**
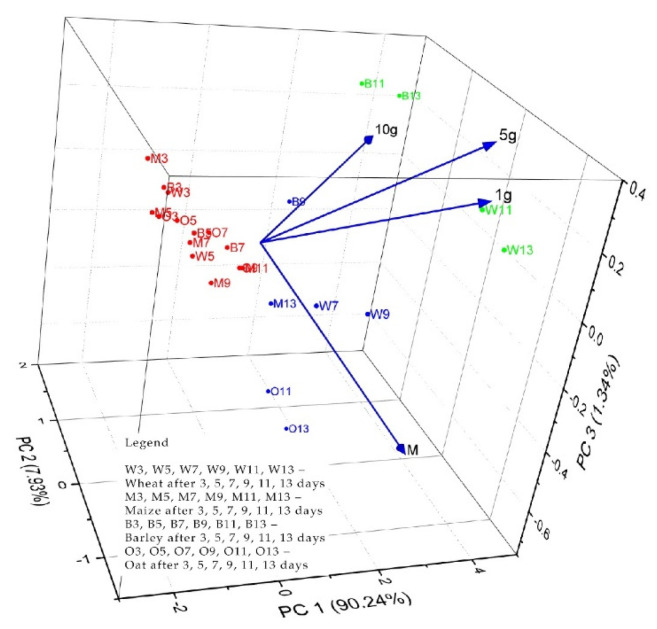
Biplot scores and loadings 3D graph for PCA analysis of plume evolution of cereal samples treated on the layer with 1 g, 5 g, and 10 g solid propolis over a period of 3, 5, 7, 9, 11, 13 days.

In Figure 10 and Figure 11, the observations and PCs obtained from the analyzed data are presented. In the biplot 3D graphs, scores, loadings PC1 vs. PC2 vs. PC3, and the formation of three groups of grain samples can be observed as follows: the first group marked in red located in the central left part of the biplot scores and loading 3D graph consists of the samples of cereals: W3, O3, M5, B3, M3, W5, M7, O5, M9, O7, B5, M11, O9, and B7 (for both cases—propolis applied on the layer and in the layer). In this classification were the treatments applied under the layer with 1 g, 5 g, and 10 g of solid propolis for a period of 3–9 days, and 11 days, in the case of the maize sample.

The second group is marked in blue and located at the bottom right of the biplot scores and loadings 3D graph and consists of the cereal samples: W7, W9, W11, W13, M13, O11, and O13. In this classification are the treatments applied under the layer with 1 g and 5 g of solid propolis for a period of 7–13 days (in the case of propolis applied under the layer), respectively, for the cereal samples M13, W7, W9, O11, and O13. In this classification are the treatments applied under the layer with 1 g and 5 g of solid propolis for a period of 7–13 days (in the case of propolis applied on the layer).

The third group, marked in green, located on the upper right side of the biplot scores and loadings 3D graph, consists of the cereal samples B9, B11 and B13. This group consists of treatments applied under the layer with 10 g of solid propolis for a period of 9–13 days and, in the case of propolis, applied on the layer from the cereal samples B11, B13, W11, and W13, which represent the treatments applied under the layer with 10 g of solid propolis for a period of 9–13 days.

**Figure 12:**
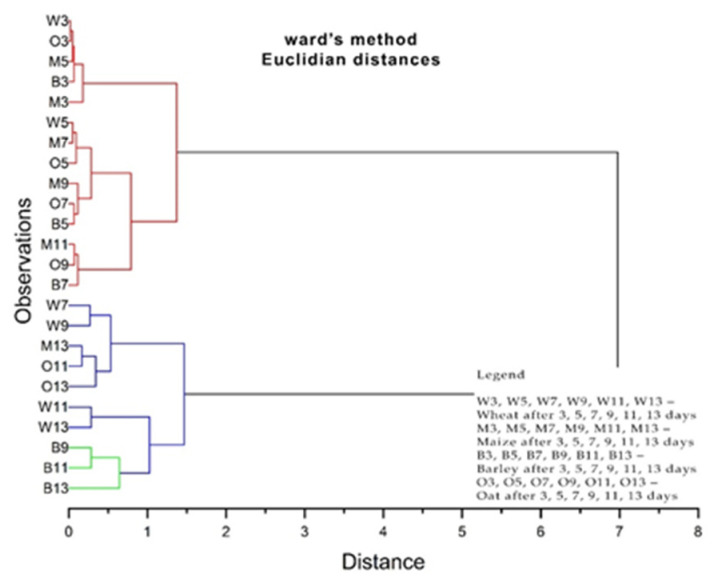
Cluster dendrogram of plume evolution of cereal samples treated under the layer with 1 g, 5 g, and 10 g of solid propolis over 3–13 days.

**Figure 13:**
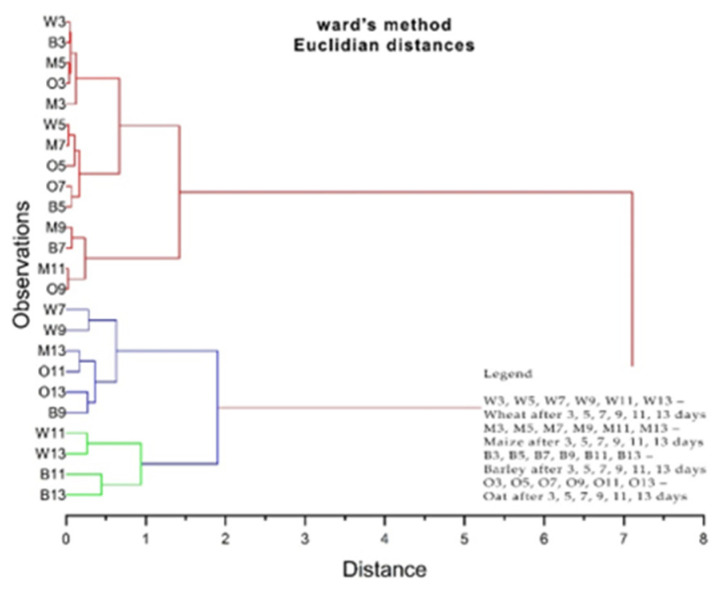
Cluster dendrogram of plume evolution of cereal samples treated on the layer with 1 g, 5 g, and 10 g of solid propolis over 3–13 days.

In hierarchical cluster analysis (HCA), samples are grouped based on similarities without considering class membership information. Compared to PCA analysis, cluster analysis (CA) uses less information, which is only the distances. It is interesting to note that based on distances alone, three groups of grain samples can be clearly distinguished, just as in the case of PCA analysis. The results obtained after the HCA are shown in Figure 12 and Figure 13, with two well-defined clusters visible.

In this case, as in the previous case, three groups of grain samples can be distinguished, exactly as in the case of the PCA analysis. The first group was marked in red, the second group was marked in blue, and the third group was marked in green.

Since all the propolis samples analyzed in this study demonstrated an antifungal effect on fungal strains commonly known to cause grain damage, this finding can serve as a basis for incorporating propolis into treatment solutions. However, given that these propolis samples also exhibited phyto-inhibitory activity, their use should be approached with caution to avoid negatively impacting cereal growth. Therefore, the doses and concentrations administered must be meticulously calculated to ensure that the beneficial effects are not offset by the phyto-inhibitory activity.

## 5. Conclusions

The benefits of propolis are derived from its different composition, which is dependent on the region in Romania. It contains significant amounts of flavonoids and phenolic compounds. The propolis samples collected from all the representative regions of Romania were characterized from a physical–chemical point of view, and the flora and plants of the area with honey potential were identified.

The inhibitory effect of the propolis samples on four types of cereals (wheat, maize, oats, and barley) has been evaluated both on the layer and under the layer. Propolis exhibited maximum inhibitory potential when applied to maize and minimum inhibitory potential when applied to wheat samples. In comparison to propolis applied under the layer, the propolis applied on the layer creates a protective film on the surface that inhibits the plume from growing. Propolis had an inhibitory effect on grain germination depending on the method of application (on the layer or under the layer), the dose applied, and the region from where it was taken.

In addition, propolis has been demonstrated to have an inhibitory impact on all strains examined, making it a useful tool in the management of specific plant illnesses and infections. The antifungal effect of propolis samples was determined on 5 strains: *A. niger*, *A. flavus*, *P. chrysogenum*, *F. oxysporum,* and *R. stolonifer*.

Propolis offers a sustainable approach to controlling cereal-crop infestations when combined with other ecological methods. In addition, it serves as an organic alternative in the management of plant crops by combating certain fungi, therefore reducing the reliance on intensive conventional practices in organic farming.

## Figures and Tables

**Table 1:** The geographical origin of the propolis samples.

Sample	Region	County of Origin	Latitude	Longitude	Landforms
S1	Transilvania	Alba	46°23′39.8″ N	22°58′06.2″ E	Mountainous
S2	Crișana	Bihor	46°41′56.0″ N	22°37′23.2″ E	Hilly
S3	Sătmar and Maramureș	Maramureș	47°32′35.2″ N	23°55′31.8″ E	Mountainous
S4	Banat	Timiș	45°42′06.5″ N	21°14′04.6″ E	Plain
S5	Oltenia	Gorj	45°10′09.3″ N	23°07′53.9″ E	Sub-mountainous
S6	Muntenia	Dâmbovița	44°58′19.6″ N	25°26′13.7″ E	Hilly
S7	Dobrogea	Constanța	43°48′25.1″ N	28°31′27.3″ E	Plain
S8	Moldova	Vaslui	46°38′01.9″ N	27°21′31.3″ E	Hilly
S9	Bucovina	Suceava	47°34′16.1″ N	25°07′01.3″ E	Mountainous

**Table 2:** Plants and flora as a predominant melliferous potential in the geographical locations of the propolis samples.

County	Cultivated Plants	Surface, ha *	Essentially Flora *	Floral Melliferous Potential (t) *
Alba	Sunflower	7636	Spruce, fir tree, beech, mountain ash, birch, acacia, blueberry, juniper, raspberry, poppies, orchards, pastures, meadow	6758
	Rapeseed and other oilseeds	3410		
	Vegetables and legumes	4686		
Bihor	Sunflower	31,750	Spruce, fir tree, mountain buckthorn, sedge, goat willow, cherry, tart cherry, apple, dandelion, Tatar maple, white mustard, sorrel, raspberry, mountain alder, juniper, blueberry and cranberry, beech, sessile oak, ash, hazelnut oak, garneau, tatar maple, and linden.	6503
	Rapeseed and other oilseeds	24,835		
	Vegetables and legumes	4774		
Maramureș	Sunflower	758	Spruce, plane tree, mountain ash, mountain alder, fir tree, larch, pine tree, yew, beech, hornbeam, elm, juniper, blueberry, cranberry, orchards, raspberry	8246.4
	Rapeseed and other oilseeds	8		
	Vegetables and legumes	1591		
Timiș	Sunflower	46,000	Fir tree, spruce, beech, oak, linden, sycamore, poplar, willow, rush, reed, water lily	4817.7
	Rapeseed and other oilseeds	10,000		
	Vegetables and legumes	318,700		
Gorj	Sunflower	596	Acacia, linden, dwarf willow, gorse, blueberry, cranberry, mountain carnation, lamb’s grass, conifers, beech, oak, chestnut	7048.7
	Rapeseed and other oilseeds	155		
	Vegetables and legumes	7150		
Dâmbovița	Sunflower	16,134	Spruce, fir tree, beech, hornbeam, sessile, turkey oak, dogwood, acacia, linden, black anise, poplar and willow	3577.4
	Rapeseed and other oilseeds	5220		
	Vegetables and legumes	11,636		
Constanța	Sunflower	74,005	Acacia, linden, oak, dwarf almonds, doves, hawthorn bushes, mint, sage, white clover, cinquefoil, sweet peas, bellflower, carnation	3002
	Rapeseed and other oilseeds	38,307		
	Vegetables and legumes	1958		
Vaslui	Sunflower	51,202	Sessile oak, pedunculated oak, hornbeam, beech, linden, field elm, boxwood, maple, ash, acacia	3395
	Rapeseed and other oilseeds	13,204		
	Vegetables	5215		
	Rapeseed and other oilseeds	4990		
Suceava	Sunflower	10,018	Spruce, fir tree, pine, aspen, linden, birch, boxwood, yew, sorrel, bluebells, foxglove, water lily, raspberry, blackberry, elder, honeysuckle, hazel bushes, strawberries, blueberry	11,256.6
	Rapeseed and other oilseeds	1600		
	Vegetables and legumes	9452		

* Data from the Romanian National Institute of Statistics and Ministry of Agriculture and Rural Development (County Directorates).

**Table 3:** Name of the cereals used to determine the phyto-inhibitory activity of propolis.

Sample	Cereal Type	Scientific Name
C1	Hexaploid bread wheat	*Triticum aestivum*
C2	Maize	*Zea mays* L.
C3	Oats	*Avena sativa* L.
C4	Barley	*Hordeum vulgare* L.

**Table 4:** Physical–chemical properties of Romanian propolis.

Sample	VO, %	Wax, %	OI, s	MP, °C	Dry Matter, %	Ash, %	Resin, %
S1	0.2 (0.02) ^b^	28.11 (1.51) ^c,d^	14.8 (3.5) ^c^	63.4 (0.2) ^c,d^	3.46 (0.22) ^a,b^	0.90 (0.05) ^b^	40.16 (1.37) ^g^
S2	0.3 (0.01) ^c^	26.55 (1.28) ^a,b,c^	13.1 (4.0) ^b^	64.1 (0.1) ^d^	3.51 (0.19) ^a,b^	0.92 (0.03) ^b^	29.93 (1.76) ^b^
S3	0.1 (0.01) ^a^	30.02 (1.54) ^d^	12.9 (3.3) ^b^	63.0 (0.2) ^b,c^	3.73 (0.27) ^a,b^	1.48 (0.07) ^d^	34.31 (1.52) ^e^
S4	0.4 (0.03) ^d^	24.54 (1.37) ^a^	13.6 (2.1) ^b^	62.2 (0.2) ^a,b^	3.27 (0.31) ^a^	2.01 (0.14) ^f^	27.05 (1.32) ^a^
S5	0.2 (0.05) ^b^	28.40 (1.16) ^c,d^	12.8 (3.3) ^b^	66.7 (0.3) ^f^	3.80 (0.26) ^b^	0.80 (0.05) ^a^	31.44 (1.15) ^c,d^
S6	0.2 (0.01) ^b^	29.08 (1.41) ^d^	10.7 (1.9) ^a^	63.5 (0.3) ^c,d^	3.74 (0.15) ^a,b^	1.06 (0.04) ^c^	30.72 (1.64) ^b,c^
S7	0.5 (0.08) ^e^	25.90 (1.09) ^a,b^	14.3 (3.6) ^c^	65.3 (0.2) ^e^	3.32 (0.20) ^a,b^	1.77 (0.09) ^e^	38.13 (2.03) ^f^
S8	0.3 (0.02) ^c^	27.77 (1.62) ^b,c^	11.0 (2.2) ^a^	62.0 (0.1) ^a^	3.66 (0.28) ^a,b^	0.79 (0.05) ^a^	29.84 (1.10) ^b^
S9	0.2 (0.04) ^b^	29.43 (1.93) ^d^	13.2 (2.7) ^b^	63.4 (0.2) ^c,d^	3.58 (0.25) ^a,b^	1.23 (0.08) ^c^	32.20 (1.01) ^d^

Abbreviations: VO—volatile oils; OI—oxidation index; MP—melting point. The numbers in the parentheses are the standard deviations of the means. Statistically significant differences between samples were compared using the *t*-test, and the data in the table with different superscript letters (a–g) in the same column indicate statistically significant differences between samples (*p* < 0.05).

**Table 5:** Bioactive composition and biological activity parameters of Romanian propolis.

Sample	TPC, mg GAE/g	TFC, mg QE/g	IC_50_, µg/mL
S1	189.4 (5.82) ^f^	84.31 (0.09) ^e^	0.333 (0.002) ^b^
S2	172.9 (3.25) ^e^	78.55 (0.08) ^c^	0.514 (0.016) ^c^
S3	152.2 (6.80) ^d^	70.10 (0.16) ^b^	0.725 (0.003) ^e^
S4	144.0 (2.09) ^c,d^	81.09 (0.98) ^d,e^	0.669 (0.010) ^d^
S5	138.2 (3.06) ^c^	77.62 (0.20) ^c^	0.884 (0.028) ^e^
S6	102.7 (2.43) ^a^	65.30 (0.11) ^a^	0.964 (0.031) ^f^
S7	189.0 (4.55) ^f^	85.19 (0.07) ^e^	0.086 (0.001) ^a^
S8	126.3 (3.14) ^b^	82.36 (0.09) ^d,e^	0.517 (0.004) ^c^
S9	150.1 (4.37) ^d^	79.54 (0.13) ^c,d^	0.615 (0.005) ^d^

Abbreviations: GAE—gallic acid equivalents; QE—quercetin equivalents; TPC—total phenolic content; TFC—total flavonoid content; IC_50_—half maximal inhibition concentration. Statistically significant differences between samples were compared using the *t*-test, and the data in the table with different superscript letters (a–f) in the same column indicate statistically significant differences between samples (*p* < 0.05).

**Table 6:** The intercorrelation matrix (Pearson, n) for correlation coefficients, r of the physico-chemical parameters of propolis.

Parameter	VO	Wax	OI	MP	Dry Matter	Ash	Resin	TPC	TFC
Wax	−0.884	-	-	-	-	-	-	-	-
OI	0.279	−0.358	-	-	-	-	-	-	-
MP	0.102	0.011	0.261	-	-	-	-	-	-
Dry matter	−0.781	0.825	−0.660	0.183	-	-	-	-	-
Ash	0.519	−0.505	0.390	−0.171	−0.662	-	-	-	-
Resin	−0.046	0.224	0.595	0.291	−0.150	−0.037	-	-	-
TPC	0.329	−0.324	0.903	0.290	−0.606	0.202	0.683	-	-
TFC	0.608	−0.575	0.624	0.077	−0.660	0.107	0.303	0.667	-
IC_50_	−0.619	0.445	−0.637	−0.072	0.693	−0.237	−0.616	−0.827	−0.792

Abbreviations: VO—volatile oils; OI—oxidation index; MP—melting point; TPC—total phenolic content; TFC—total flavonoid content; IC_50_—half maximal inhibition concentration.

**Table 7:** Flavonoids: quercetin and rutin concentrations in the propolis aqueous extracts.

Sample	Quercetin (mg/mL); RSD%	Rutin (mg/mL); RSD%
S1	0.57; 1.66 ^a^	0.0143; 1.47
S2	0.62; 2.80 ^a,b^	0.0127; 1.30
S3	0.65; 2.83 ^a^	0.0093; 2.03
S4	0.74; 2.94	0,0102; 2.24
S5	0.71; 2.79	0.0134; 1.31
S6	0.78; 2.85	0.0119; 1.19
S7	0.66; 2.44	0.0185; 1.02
S8	0.81; 1.91	0.0168; 1.55
S9	0,59; 1.72 ^a^	0.0150; 1.96

Statistically significant differences between samples were compared using the *t*-test, and the data in the table with different superscript letters (a–f) in the same column indicate statistically significant differences between samples (*p* < 0.05).

**Table 8:** Physical characteristics of the cereals used.

Sample	Relative Weight of 1000 Seeds, g	Absolute Mass of 1000 Seeds, g	Moisture, %	Hectoliter Mass, kg/hL	Glassiness, %
C1	40	35	13.8	77.1	91
C2	169	287	14.4	73.8	88
C3	25	23	12.9	41.1	-
C4	41	60	14.2	63.7	-

**Table 9:** The antimicrobial effect of the aqueous extracts of propolis against selected fungal strains.

Sample No.	Strain				
	*A. niger*	*A. flavus*	*P. chrysogenum*	*F. oxysporum*	*R. stolonifer*
S1	23.83 ± 0.29	26.33 ± 0.29	21.67 ± 0.58	27.77 ± 1.14 *	25.5 ± 0.50
S2	18.67 ± 0.58	17.00 ± 0.00	17.16 ± 0.29	23.67 ± 0.58	20.00 ± 0.00
S3	16.17 ± 0.29	20.17 ± 0.29	18.67 ± 0.58 *	26.50 ± 1.73 *	22.17 ± 0.76
S4	15.67 ± 0.57	18.00 ± 0.87	16.83 ± 0.29	22.00 ± 0.00	24.33 ± 0.58
S5	16.67 ± 1.04	21.67 ± 1.44 *	18.00 ± 0.00	25.00 ± 0.00	19.17 ± 0.29
S6	18.17 ± 0.29	19.00 ± 0.00	16.17 ± 0.29	22.67 ± 0.58	23.00 ± 4.33 *
S7	17.50 ± 0.50	17.00 ± 0.87	18.67 ± 0.58 *	26.33 ± 0.58	20.00 ± 0.00
S8	18.00 ± 0.00	21.33 ± 0.58	17.00 ± 0.00	24.00 ± 0.00	25.00 ± 0.00
S9	16.50 ± 0.50	18.00 ± 0.00	17.67 ± 0.58 *	21.67 ± 0.58	21.00 ± 3.50 *
Voriconazole 1 μg	45.00 ± 0.00	43.00 ± 0.00	18.00 ± 0.00	29.00 ± 0.00	16.00 ± 0.00

Note: Values are means of 3 independent experiments ± standard deviation; * The *p*-values of Student’s *t*-test indicate no significant difference at *p* > 0.05.

**Table 10:** Pearson correlation of TCP and TFC of propolis samples with the MIC of the tested microorganism.

Tested Microorganism (MIC)	Pearson Correlation With:			
	TPC	*p*-Value	TFC	*p*-Value
*A. niger*	0.438	0.238	0.667	0.050
*A. flavus*	0.109	0.781	0.155	0.691
*P. chrysogenum*	0.731	0.025	0.435	0.242
*F. oxysporum*	0.600	0.088	0.667	0.050
*R. stolonifer*	−0.164	0.674	0.096	0.050

Significantly correlated at *p*-value ≤ 0.05.

## Data Availability

Data are contained within the article.

## Supplementary Materials

The following supporting information can be downloaded at: https://www.mdpi.com/article/10.3390/plants13233355/s1, Table S1. Retention times, area under the curve and percentage area of of propolis active substances of S1 (Alba County) aqueous propolis extract. Tables S2–S9: Phyto-inhibitory activity of propolis samples.
