# Supplementary material for: Inhibitory Effects and Composition Analysis of Romanian Propolis: Applications in Organic and Sustainable Agriculture
Source: Plants (Basel). 2024 Nov 29;13(23):3355. doi: 10.3390/plants13233355 (PMC11644543; doi:10.3390/plants13233355)
Supplement: Supplementary file 1 [file plants-13-03355-s001.zip › plants-3318732-supplementary.pdf]

Contents

Supplementary Table S1. Retention times, area under the curve and percentage area of of propolis active substances of S1 (Alba County) aqueous propolis extract

**Peak number identification**	**Retention time (min)**	**Area (AUC)**	**Area (%)**	**Compound**
1	2.003	1699.06	3.94	Rutin
2	2.55	1.46E+04	34.16	Caffeic acid
3	2.862	1.76E+04	40.71	*p*-Coumaric acid
4	3.176	1028.103	2.44	3,4-Dimethoxycinnamic acid
5	3.314	630.5867	1.47	Quercetin (ketonic form)
6	3.427	2628.946	6.04	Quercetin (enolic form 1)
7	3.611	561.3952	1.33	Quercetin (enolic form 2)
8	3.92	290.008	0.65	Unidentified
9	4.178	422.0375	0.87	Apigenin
10	4.376	443.5021	1.05	Unidentified
11	4.533	776.6244	1.92	Kaempferol
12	4.681	1346.375	3.10	Unidentified
13	5.568	53.63338	0.14	Unidentified
14	5.725	250.9789	0.57	Unidentified
15	6.441	233.4592	0.59	Unidentified
16	7.6366	169.9313	0.35	Unidentified
17	7.969	56.34415	0.21	Galangin
18	8.29	89.54768	0.20	Caffeic acid phenethyl ester
19	9.307	92.80241	0.20	Cinnamic acid ester
20	9.522	36.10306	0.09	Unidentified

Abbreviation: AUC - area under the curve

Phyto-inhibitory activity of propolis samples: **Supplementary Table S2-S9**

Supplementary Table S2. Plumule growth lengths (mm) for wheat samples treated with different amounts of propolis powder under the layer (C1a) in time.

Day	*1 g*									*5 g*									*10 g*									M [mm]
	S1	*S2*	*S3*	*S4*	*S5*	*S6*	*S7*	*S8*	*S9*	*S1*	*S2*	*S3*	*S4*	*S5*	*S6*	*S7*	*S8*	*S9*	*S1*	*S2*	*S3*	*S4*	*S5*	*S6*	*S7*	*S8*	*S9*	
3	16	16	14	15	15	14	13	15	12	7	5	5	3	4	6	5	6	6	0	0	0	0	0	0	0	0	0	24
5	32	30	31	30	33	31	30	33	29	19	18	15	12	13	16	14	17	16	4	3	4	2	3	2	4	3	2	39
7	80	78	77	77	81	79	76	79	75	55	52	50	51	49	53	54	53	52	18	15	13	10	12	13	14	11	9	82
9	104	98	96	93	107	99	97	100	92	66	64	63	62	60	61	65	61	63	25	24	20	21	22	24	25	23	21	101
11	117	110	113	112	120	116	111	119	109	101	98	96	95	89	90	97	94	98	32	30	25	25	26	28	33	27	26	123
13	138	125	122	120	131	128	125	133	121	113	113	115	111	112	114	116	113	115	41	40	38	36	37	39	42	38	35	145

Supplementary Table S3. Plume growth lengths (mm) for wheat samples treated with different amounts of propolis powder per layer (C1b) in time.

Day	*1 g*									*5 g*									*10 g*									M [mm]
	S1	*S2*	*S3*	*S4*	*S5*	*S6*	*S7*	*S8*	*S9*	*S1*	*S2*	*S3*	*S4*	*S5*	*S6*	*S7*	*S8*	*S9*	*S1*	*S2*	*S3*	*S4*	*S5*	*S6*	*S7*	*S8*	*S9*	
3	10	12	11	12	11	10	10	11	12	4	2	3	1	3	3	3	4	2	0	0	0	0	0	0	0	0	0	24
5	23	21	20	24	22	24	25	22	20	8	10	7	8	5	8	9	9	7	0	1	2	0	1	0	2	0	0	39
7	69	66	64	65	64	60	61	67	75	40	41	39	43	37	44	46	42	40	10	8	7	5	6	8	9	4	5	82
9	92	85	84	81	92	88	84	100	92	52	51	54	50	48	52	55	51	53	15	16	11	12	14	13	17	15	10	101
11	102	100	103	101	109	104	99	105	109	101	98	96	95	89	90	97	94	98	24	26	19	20	17	20	23	21	18	123
13	124	116	113	111	120	119	115	120	110	113	107	104	102	98	108	105	101	107	33	32	29	27	29	33	37	30	24	145

Supplementary Table S4. Plume growth lengths (mm) for maize samples treated with different amounts of propolis powder under the layer (C2a) in time.

Day	*1 g*									*5 g*									*10 g*									M [mm]
	S1	*S2*	*S3*	*S4*	*S5*	*S6*	*S7*	*S8*	*S9*	*S1*	*S2*	*S3*	*S4*	*S5*	*S6*	*S7*	*S8*	*S9*	*S1*	*S2*	*S3*	*S4*	*S5*	*S6*	*S7*	*S8*	*S9*	
3	3	2	1	2	1	1	2	1	0	0	0	0	0	0	0	0	0	0	0	0	0	0	0	0	0	0	0	15
5	14	14	14	12	13	14	12	11	11	2	2	1	0	1	1	0	1	0	0	0	0	0	0	0	0	0	0	23
7	28	26	27	24	27	27	24	25	23	21	17	12	10	19	12	16	14	11	4	2	0	1	3	1	2	0	0	37
9	35	33	31	30	33	34	32	30	28	28	24	22	18	27	23	20	21	19	12	10	8	9	11	10	10	8	9	54
11	40	38	40	41	40	39	41	40	37	38	33	32	27	35	37	39	33	28	18	18	15	17	16	15	16	14	13	65
13	53	51	54	52	52	53	50	51	50	49	48	40	34	49	50	48	46	37	23	24	20	21	22	20	21	19	19	83

Supplementary Table S5 Plume growth lengths (mm) for maize samples treated with different amounts of propolis powder on the layer (C2b) in time.

Day	*1 g*									*5 g*									*10 g*									M [mm]
	S1	*S2*	*S3*	*S4*	*S5*	*S6*	*S7*	*S8*	*S9*	*S1*	*S2*	*S3*	*S4*	*S5*	*S6*	*S7*	*S8*	*S9*	*S1*	*S2*	*S3*	*S4*	*S5*	*S6*	*S7*	*S8*	*S9*	
3	1	1	0	0	0	0	1	0	0	0	0	0	0	0	0	0	0	0	0	0	0	0	0	0	0	0	0	15
5	4	5	4	3	3	4	4	3	2	0	1	0	0	0	0	0	0	0	0	0	0	0	0	0	0	0	0	23
7	21	18	22	15	23	20	19	18	16	13	11	7	6	12	5	9	8	10	4	0	0	0	1	0	1	0	0	37
9	28	26	25	24	27	26	29	25	27	22	17	15	11	17	18	13	15	16	12	6	6	7	9	7	7	5	7	54
11	34	31	33	37	35	34	36	36	32	30	25	27	23	28	31	33	29	24	18	15	11	12	13	11	11	10	9	65
13	47	45	49	46	43	42	44	46	45	37	42	35	29	33	44	40	41	32	21	20	18	17	20	19	16	15	14	83

Supplementary Table S6. Plume growth lengths for oat samples treated with different amounts of propolis powder under the layer (C3a) in time.

Day	*1 g*									*5 g*									*10 g*									M [mm]
	S1	*S2*	*S3*	*S4*	*S5*	*S6*	*S7*	*S8*	*S9*	*S1*	*S2*	*S3*	*S4*	*S5*	*S6*	*S7*	*S8*	*S9*	*S1*	*S2*	*S3*	*S4*	*S5*	*S6*	*S7*	*S8*	*S9*	
3	16	15	18	19	15	17	16	16	14	5	5	4	5	4	4	5	4	3	0	0	0	0	0	0	0	0	0	25
5	24	22	24	24	23	25	23	22	22	11	12	10	11	10	12	11	11	9	4	2	2	1	3	2	2	1	1	30
7	33	32	33	32	32	31	33	32	30	21	22	20	22	21	21	22	21	20	12	11	9	8	11	10	9	8	7	44
9	42	41	40	42	41	40	41	40	38	37	39	32	38	36	37	35	34	31	23	22	22	21	22	21	20	21	20	65
11	51	55	56	52	52	54	53	53	50	45	44	48	42	47	46	43	41	42	30	27	28	23	30	27	29	26	25	102
13	60	62	63	59	61	62	61	60	59	58	50	53	52	53	52	51	50	50	39	36	33	38	38	36	36	37	34	123

Supplementary Table S7. Plume growth lengths for oat samples treated with different amounts of propolis powder on the layer (C3b) in time.

Day	*1 g*									*5 g*									*10 g*									M [mm]
	S1	*S2*	*S3*	*S4*	*S5*	*S6*	*S7*	*S8*	*S9*	*S1*	*S2*	*S3*	*S4*	*S5*	*S6*	*S7*	*S8*	*S9*	*S1*	*S2*	*S3*	*S4*	*S5*	*S6*	*S7*	*S8*	*S9*	
3	9	7	5	8	7	9	8	7	6	2	1	0	2	0	1	2	0	0	0	0	0	0	0	0	0	0	0	25
5	15	13	16	15	14	18	13	15	14	6	5	4	7	3	8	5	4	3	0	0	0	0	0	0	0	0	0	30
7	22	23	20	24	25	20	26	28	21	18	17	15	16	15	17	17	16	14	5	5	4	4	5	5	5	4	3	44
9	31	30	30	33	32	34	37	35	30	29	31	27	29	25	28	26	27	26	23	11	13	10	12	12	11	14	11	65
11	46	51	50	48	49	50	47	46	45	38	37	40	39	40	38	36	35	33	30	24	23	18	26	22	25	21	20	102
13	53	56	61	55	56	58	57	54	53	58	44	47	45	46	47	42	43	41	35	31	28	34	34	32	33	30	30	123

Supplementary Table S8. Plume growth lengths (mm) for barley samples treated with different amounts of propolis powder under the layer (C4a) in time.

Day	*1 g*									*5 g*									*10 g*									M
	S1	*S2*	*S3*	*S4*	*S5*	*S6*	*S7*	*S8*	*S9*	*S1*	*S2*	*S3*	*S4*	*S5*	*S6*	*S7*	*S8*	*S9*	*S1*	*S2*	*S3*	*S4*	*S5*	*S6*	*S7*	*S8*	*S9*	[mm]
3	14	12	10	11	13	11	12	11	11	10	10	8	7	11	10	9	8	8	0	0	0	0	0	0	0	0	0	23
5	29	28	29	26	27	26	27	25	26	18	15	17	16	17	16	16	17	14	12	11	10	8	11	11	10	9	9	40
7	38	33	37	34	37	35	39	36	34	29	31	30	27	29	30	28	27	28	20	18	16	19	18	17	19	17	16	55
9	64	66	61	60	65	66	62	64	61	56	55	54	50	53	52	51	51	49	50	52	50	47	52	51	50	47	49	90
11	88	85	83	81	87	83	86	84	82	79	75	77	71	76	74	72	78	66	63	62	56	51	59	60	58	56	53	101
13	110	105	108	107	106	108	105	103	103	91	90	88	89	90	90	87	89	87	80	84	77	73	83	82	80	79	78	132

Supplementary Table S9. Plume growth lengths for barley samples treated with different amounts of propolis powder on the layer (C4b) in time.

Day	*1 g*									*5 g*									*10 g*									M
	S1	*S2*	*S3*	*S4*	*S5*	*S6*	*S7*	*S8*	*S9*	*S1*	*S2*	*S3*	*S4*	*S5*	*S6*	*S7*	*S8*	*S9*	*S1*	*S2*	*S3*	*S4*	*S5*	*S6*	*S7*	*S8*	*S9*	[mm]
3	7	6	6	7	7	5	7	6	6	6	5	3	4	5	5	4	4	3	0	0	0	0	0	0	0	0	0	23
5	22	19	20	18	20	21	22	17	19	12	10	11	10	10	12	11	11	9	6	5	6	3	5	6	5	3	2	40
7	31	25	29	27	31	28	33	30	27	29	24	23	21	22	21	20	20	22	20	9	9	7	8	8	9	6	5	55
9	51	55	50	52	53	54	52	57	51	56	44	45	38	44	43	42	41	37	50	33	38	30	41	39	37	35	36	90
11	79	77	72	73	76	75	78	72	71	79	69	68	60	65	64	63	67	61	63	50	44	40	48	49	51	47	42	101
13	102	98	99	94	95	100	93	97	90	91	82	79	77	81	83	78	78	76	80	71	66	62	65	68	69	67	70	132
